# How Do Glucocorticoids Used in Rheumatic Disease Affect Body Weight? A Narrative Review of the Evidence

**DOI:** 10.1002/acr.23879

**Published:** 2020-03-27

**Authors:** Catharine Morgan, Ruth E. Costello, David W. Ray, William G. Dixon

**Affiliations:** ^1^ University of Manchester Manchester UK; ^2^ Manchester Academic Health Science Centre and The University of Manchester Manchester UK; ^3^ University of Manchester, Manchester, and University of Oxford Oxford UK

## Abstract

Glucocorticoids (GCs) are widely used to effectively treat inflammatory disease, but GCs have a number of recognized side effects. Patients and clinicians view these side effects differently, with clinicians most concerned with serious side effects such as osteoporosis and diabetes mellitus. Consequently, these side effects are well researched with clinical guidelines and recommendations. A side effect of particular concern to patients is weight gain, but this topic has not been well researched, and consequently clinicians find it difficult to provide patients with accurate information about the potential of weight gain. The aim of this review is to provide an overview of GC use specifically in rheumatic disease, patient views on GC therapy, and GC‐induced weight gain. We will discuss the evidence, including the extent and the impact of weight gain on the patient, and highlight areas that warrant further investigation.

## Introduction

Since their introduction in 1948 1, glucocorticoids (GCs), or steroids, have been widely used to treat inflammatory disease. Despite their clinical effectiveness, there are many recognized adverse effects of concern to both patients and clinicians. Informed treatment decisions by patients and clinicians are based on the balance of GC benefits and harms 2, 3. These decisions require information on the probability and nature of the benefits and harms (e.g., the onset, duration, and reversibility of the adverse effect) and are subject to a value judgement, a construct of how important the outcome is to the individual 4. While clinicians may view certain adverse events as nonserious and thus of less importance, patients may judge these differently. Patient attitudes toward GC‐associated adverse effects have previously been shown to differ from those of clinicians 5. One side effect of particular concern to patients is weight gain 6, 7. In clinical practice, patients commonly decline GCs because of concerns about weight gain, even when potential benefits are high and clinicians believe the benefit/harm balance is favorable.

**Figure 1:**
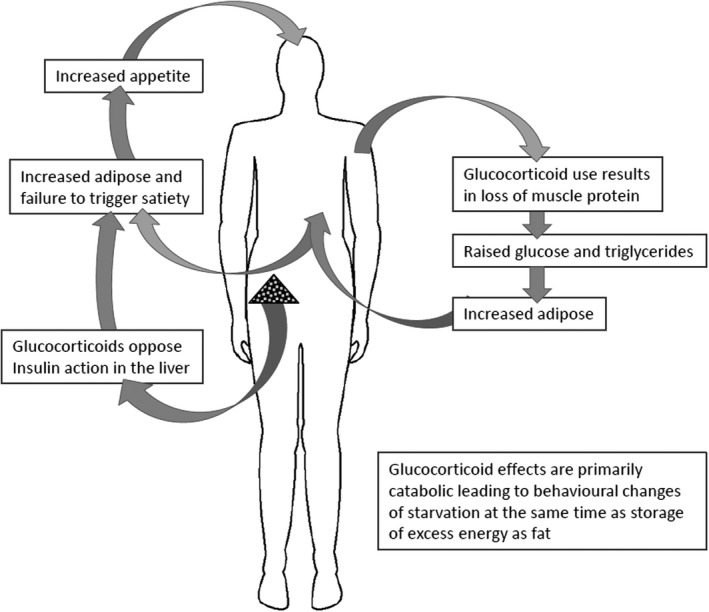
Impact of glucocorticoids on body composition.

The impact of GCs on body composition can be profound. In humans, this impact results in central deposition of adipose tissue, with a marked catabolic effect on bone and muscle. A number of mechanisms are proposed, with the final impact being determined by the combination of these (Figure 1). The actions of GCs include an increase in appetite, insulin resistance at the liver, which impairs effective management of excess calorie intake and promotes a liver lipogenic program, and catabolic actions on bone and muscle, which mobilize amino acids for gluconeogenesis in the liver. Additionally, relevant actions include suppression of the reproductive axis, resulting in sex steroid deficiency, which further impacts muscle mass and function. The GC dose relationship to these effects is complex, reflecting the large variation in GC sensitivity seen in individuals. In a systematic review, a dose‐response relationship of oral GCs was not found on energy intake, appetite, and body weight or body composition. The authors suggested that duration was important, with short‐term therapy having small increases in energy intake but not in weight gain, and longer‐term GC therapy resulting in clinically significant weight gain 8. Adverse effects are thought to be more significant at daily dose‐equivalent exposures of >5 mg prednisolone 9, with adverse effect risk appearing to rise as an exponential to the daily GC dose 10, 11.

In this review, we provide an overview of GC use specifically in rheumatic disease, patient views on GC therapy, and GC‐induced weight gain. We discuss the evidence found during our literature search (for search strategy, see Supplementary Appendix A, available on the *Arthritis Care & Research* web site at http://onlinelibrary.wiley.com/doi/10.1002/acr.23879/abstract), including the extent and the impact of weight gain on the patient. We highlight areas that warrant further investigation to aid informed treatment decisions, for both the clinician and patient, regarding weight gain.

## GC utilization in rheumatic disease

GCs are an effective treatment for various inflammatory and autoimmune disorders. An estimated 0.85% of the adult general population have received oral GCs at some time and 0.75% are prescribed GCs for at least 3 months 12. Respiratory disease and rheumatic diseases were the most frequently recorded indications for both long‐term and overall GC therapy 12, 13. GC therapy prescribed for rheumatic conditions for <30 days is uncommon 14. Specifically for inflammatory musculoskeletal conditions, there are reports of up to 2 in every 3 patients with rheumatoid arthritis (RA) ever using GCs 15, 16, 17. Current guidelines from the European League Against Rheumatism (EULAR) still advocate their use in early RA because of their powerful and rapid efficacy 18. Uptake in systemic lupus erythematosus exceeds 70% 19, with near universal use in conditions such as vasculitis and polymyalgia rheumatica 20. This widespread use of GC therapy reflects their efficacy. However, GCs are also well known for a range of adverse effects associated with dose, duration, and timing, including osteoporosis and fracture, infection, diabetes mellitus, cataracts and glaucoma, weight gain, adrenal insufficiency, skin changes, and cardiovascular disease. These effects have been reviewed widely elsewhere 21. The safety of certain GC‐associated adverse events is far more widely studied and understood than for others. For example, there is much research investigating GC‐induced osteoporosis and fractures, supporting numerous clinical guidelines and recommendations (American College of Rheumatology recommendations 22; National Institute for Health and Clinical Excellence guidelines). Yet for other events such as weight gain and insomnia, 2 of the side effects that patients are most concerned about 6, 7, much less is known. So how do patients currently view GC treatment and this potential side effect in the absence of good evidence?

## Patient beliefs about GC treatment

Evidence about patient beliefs toward medication shows patients express a high level of perceived need for GC treatment but also a high level of concern for treatment consequences 23, 24, 25, 26. A survey of GC users in France found 86% considered GCs to be efficient and 68% considered GCs unsafe 24. Of 98 systemic sclerosis patients, 73% showed concern for adverse consequences, with 82% worried about the long‐term effects of GCs 26.

Larger clinical trials show low withdrawal rates due to reasons other than adverse medication effects but rarely include further details 27, 28, 29, 30. Both in the Circadian Administration of Prednisolone in Rheumatoid Arthritis trial 26, and in the more recent Giant‐Cell Arteritis Actema trial 30, patient withdrawals did not include detailed reasons. Bakker at al 29 reported that of 108 of the 347 patients starting the Computer Assisted Management of Rheumatoid Arthritis (CAMERA) trial, 80% withdrew because of potential GC use and 20% due to time constraints. In the West of Scotland Early Rheumatoid Arthritis Corticosteroid Trial (WOSERACT), a randomized clinical trial (RCT) of low‐dose prednisolone compared to placebo, of 247 RA patients eligible, 19% (46 of 247) chose not to take part in the trial due to concerns over the prospect of taking GCs 28. A cross‐sectional survey conducted in 2 WOSERACT outpatient clinics in Glasgow investigated attitudes toward GC treatment. Researchers found that 68% of patients (100 of 148) with RA were unwilling to be treated with oral GCs, and when asked to list known side effects, weight gain was the side effect listed most often by respondents 31.

## Patient beliefs about weight gain

Patients’ concerns about GC treatment have been shown to be different from those of clinicians. In a study from 2010, the risk of osteoporosis, cardiovascular disease, and diabetes mellitus were ranked in the top 5 most worrisome adverse effects by both patients and clinicians. Weight gain was ranked the fourth most worrisome adverse effect by patients but only the sixth most worrisome for clinicians 5. As shown in a cross‐sectional study investigating attitude toward GC use, GC‐naive patients considered weight gain as a major side effect, more so than osteoporosis (78% [116 of 148] versus 10% [15 of 148]) 31. A recent survey of online health community users in the UK who reported GC use found that weight gain was the side effect most important to users 7. Similarly, in a survey of patients with adrenal insufficiency from 5 European countries, over 50% of respondents reported significant concerns about weight gain 32. A qualitative study of patients with antineutrophil cytoplasmic antibody–associated vasculitides from the UK, Canada, and the US who used GCs also found that weight gain and change in appearance were widely described as salient side effects across all countries 33.

Further evidence from social media indicates concerns from GC‐treated patients about weight gain that may otherwise be unreported. PatientsLikeMe is a patient‐powered network where patients contribute health and well‐being information in real time to inform other patients and aid research. At the time of writing (March 2018), of all prednisone‐treated patients, irrespective of indication (n = 4,950), the most commonly reported side effects were weight gain (n = 435) and increased appetite (n = 108). In patients with RA only (n = 271), weight gain (n = 85) and increased appetite (n = 21) were again the most commonly reported adverse effects (https://www.patientslikeme.com/treatments/show/139-prednisone-side-effects-and-efficacy?brand=f). Analysis of public discussions on social media platforms such as Facebook and Twitter can also be informative and has proven a useful tool to support pharmacovigilance by highlighting patterns not seen in spontaneous reporting from clinicians 34. In a recent study of Twitter posts mentioning prednisolone or prednisone, insomnia and weight gain were the most commonly discussed adverse events 6.

This evidence supports the idea that weight gain is a common side effect of importance to patients and influences patient decision‐making. Accurately describing to patients when this weight gain may occur would be valuable, as well as discussing for how long and whether weight gain returns to baseline following GC cessation and at what rate. The following sections describe the current evidence.

## Probability and extent of weight gain

When reviewing the literature on the extent of weight gain in GC‐treated RA populations and indeed for other nonrheumatic disease populations 8, the extraction of this outcome and comparability is difficult between studies, with some reporting mean weight gain 28, 29, 35, 36, 37, 38 or others reporting the proportion of individuals experiencing weight gain 35, 39 or weight gain in terms of total fat mass 40. In the group of RCTs investigating the efficacy of GCs, reporting weight gain as an adverse event over a similar time period, weight gain findings were inconsistent. A mean weight change ranging between no change to a weight gain of 5 kgs was reported in GC‐treated groups, compared to no change to a 3 kg weight gain in the untreated groups over 1–2 years 28, 29, 35, 36, 37, 38 (Table 1). Two further RCTs reported the proportion of patients with weight gain. In the active treatment group, 4 of 80 gained weight, and none had weight gain in the placebo group 35. In another, 8 of 98 patients in the treatment arm gained weight 39, with both studies omitting to report the extent of weight gain. One study as part of the larger multicenter Combinatietherapie Bij Reumatoïde Artritis (COBRA) light trial 40 reported that in the GC‐treated group of 38 prednisolone‐naive early RA patients total body mass increased by 1.6 kg and total fat mass by 1.3 kg. At 26 weeks, the prevalence of overweight and obesity increased to 50% and 13%, respectively.

**Table 1:** Studies reporting weight changes^a^

Study type	Author, year (ref.)	Population	Aim	No.	Prednisolone exposure	Length of follow‐up	Mean weight change at end of follow‐up	How weight was measured
RCT	Kirwan, 1995 36	RA, duration <2 years	Efficacy of GCs	Prednisolone (n = 61), placebo (n = 67)	7.5 mg; low dose	2 years	No significant increase in weight	Measured at each study visit
RCT subanalysis of Kirwan, 1995	Hickling, 1998 45	RA, duration <2 years	To report response to prednisolone discontinuation	Prednisolone (n = 36), placebo (n = 39)	7.5 mg; low dose	3 years	By 3 years prednisolone group lost 3.1 kg (95% CI 1.9, 4.3) vs. placebo 1.5 kg (95% CI 0.3, 2.7)	Measured at each study visit
RCT	Boers, 1997 38	RA, duration <2 years	Efficacy of GCs	Prednisolone (n = 77), placebo (n = 79)	60 mg tapered to 7.5 mg at week 6 until week 28 when prednisolone was stopped; high dose	56 weeks	Weight increase at 28 weeks: significant difference between groups: 2.5 kg (95% CI 1.8, 3.2) in GC group and 0.7 kg (95% CI –0.2, 2.2) in placebo group; weight gain at 56 weeks: no significant difference	Measured at each study visit
RCT	Wassenberg, 2005 35	RA, duration <2 years	Efficacy of GCs	Prednisolone (n = 80), placebo (n = 86)	5 mg; low dose	2 years	Prednisolone +5 kg; placebo +0.3 kg	Measured at each study visit
RCT	Van Everdingen, 2002 37	RA, duration <1 year		Prednisolone (n = 40), placebo (n = 41)	10 mg; low dose	2 years	Prednisolone significant increase from 77 ± 19 kg to 80 ± 20 kg; placebo no significant change	Measured at each study visit
RCT	Capell, 2004 28	RA, duration <3 years	Efficacy of GCs	Prednisolone (n = 84), placebo (n = 83)	7 mg; low dose	2 years	Prednisolone 4 kg; placebo 3 kg	Measured at each study visit
RCT	Bakker, 2012 29	RA, duration <1 year	Efficacy of GCs	Prednisolone (n = 117), placebo (n = 119)	10 mg; low dose	2 years	Prednisolone 2.9 ± 4.2 kg; placebo 1.3 ± 5.3 kg	Measured at each study visit
RCT	Wung, 2008 42	Severe GPA	To assess the quantity, duration, and progression of weight change in patients who received GCs for active GPA under WGET protocol	n = 157, all used prednisone	1 mg/kg/day tapered to nothing over 12 weeks, restarted if disease flares; low dose	1 year	3.9 ± 6.9 kg (4.4% increase), 38 patients (24%) gained ≥10 kg	Measured at each study visit
RCT substudy	Konijn, 2016 40	Early RA	To investigate effect of high‐down and step‐down prednisolone regimens on body composition	n = 108; n = 38 with DXA scan at start of treatment	Prednisolone 60 mg/day, tapered to 7.5 mg/day in 6 weeks; MTX and SSZ; prednisolone 30 mg/day, tapered to 7.5 mg/day in 8 weeks and MTX; high dose	26 weeks	Total body mass increase 1.6 kg; total fat mass increase 1.3 kg; BMI increased from 25.6 to 26.2 in glucocorticoid‐treated patients; prevalence of overweight increase and obesity relative to baseline	Measured at baseline (before or soon after treatment) and after 26 weeks
RCT	Verschueren, 2017 39	Early RA	To compare the effectiveness of different initial csDMARD combinations, with or without GC remission 52 weeks after treatment initiation	High risk n = 289: COBRA classic MTX, SSZ + weekly step down prednisolone n = 98; COBRA slim: MTX + weekly step down prednisolone n = 98; COBRA avant‐garde: MTX, LEF + weekly step down prednisolone (30–5.5 mg) n = 93; low risk n = 90, COBRA slim n = 43, MTX no GC n = 47	COBRA classic: 60–7.5 mg, COBRA slim and COBRA avant‐garde: 30–5.5 mg; high dose	2 years	Not given, only number with weight gain	Patients asked about AEs at each visit, including weight gain
Cohort	Pincus, 2013 44	RA	Analysis of prednisolone treatment over 25 years	n = 290	Various	25 years	2.7 kg in those monitored 1 year or less; no change if monitored for >1 year	Measured at clinic visits
Cohort	Curtis, 2006 11	GC users	Assessing prevalence of adverse events, dose, and duration dependence	n = 2,167	Various	18 months	60–80% reported weight gain; weight gain increased with increased cumulative dose	Self‐reported
Register	Huscher, 2009 41	RA	Patterns relating frequency of adverse effects to dosage and duration	GC use for >6 months (n = 472); no GC use in past 12 months (n = 307)	Various	12 months	No GC use: 9.5% reported weight gain; GC users: <5 mg/day, 8.7%; 5–7.5 mg/day, 22.4%; and >7.5 mg/day, 21.3% reported weight gain	Self‐reported

a RCT = randomized clinical trial; RA = rheumatoid arthritis; GC = glucocorticoid; 95% CI = 95% confidence interval; GPA = granulomatosis with polyangiitis; WGET = Wegner's Granulomatosis Etanercept Trial; DXA = dual x‐ray absorptiometry; MTX = methotrexate; SSZ = sulfasalazine; BMI = body mass index; csDMARD = conventional synthetic disease‐modifying antirheumatic drug; COBRA = Combinatietherapie Bij Reumatoïde Artritis trial; LEF = leflunomide.

The comparison of studies on weight gain and GC use is further complicated by the complex nature of the dose and duration of GC use and how this usage is measured and reported. Most of the RCT examples above used GC doses varying from 5 mg to 10 mg, where weight gain in the treated group (prednisolone exposure of 7.5 mg, considered a low dose), ranged from no weight gain 29 to 4 kg (26) and 5 kg (35). The RCTs using a dose of prednisolone higher than 10 mg indicated weight gain of mean ± SD 2.9 ± 4.2 kg 35 and change of weight from mean ± SD 77 ± 19 kg to 80 ± 20 kg 34. Those studies tapering from 60 mg to 7.5 mg, considered a high dose of prednisolone, showed weight increases of 2.5 kg (95% confidence interval [95% CI] 1.8, 3.2) 37 and mean ± SD body mass index change from 25.7 ± 4.0 kg/m² to 26.3 ± 4.2 kg/m² 40 (Table 1). There was no clear dose‐response evidence from RCTs, although across all doses there were significant weight increases. The greatest difference between groups was 4.7 kg in the study of 5 mg prednisolone versus placebo over a 2‐year period 35 (Table 1).

Cohort studies include patients on a wide range of dosages and, in theory, thus allow comparison between different real‐world dosages and their impact on weight gain. There is some evidence from observational research to suggest a dose‐response relationship. A study of patients with RA found increased frequency of reported weight gain at higher doses, with ~20% of patients reporting weight gain at prednisolone doses of 5 mg/day or more versus <10% reporting weight gain at <5 mg/day or no prednisolone in the past 12 months 41. Curtis et al 11 reported that in long‐term users of GCs with a variety of conditions, there were significantly increased odds of reporting weight gain, at cumulative doses >1.7 grams compared to cumulative doses <1.7 grams, with odds ratios (ORs) increasing with higher cumulative doses compared to <1.7 grams, from OR 1.42 (95% CI 1.08, 1.85) at 1.7–2.8 grams to OR 2.20 (95% CI 1.65, 2.95) at >4.7 grams. One significant challenge in observational analyses is that GC treatment is often dynamic, with dosages changing through time, and weight is measured at infrequent and sporadic intervals. Few statistical models are yet able to consider the impact of time‐varying exposure on a continuous outcome through time. While RCTs commonly study a fixed dose and collect outcome data at fixed intervals, thereby potentially allowing the examination of trajectories of weight gain, they often only report weight gain from baseline to the end of the study.

## Time scale of weight gain and loss

The timescale and pattern of weight gain following GC exposure is not well defined in the literature. In clinical trials, those patients reporting a 5‐kg gain over 2 years could, for example, represent a gain in the first month then plateau, or they could be indicative of a steady increase over the 2‐year period. In a cohort study, Curtis et al 11 found that a high percentage of long‐term GC users reported very bothersome weight change at all quartiles of cumulative prednisone‐equivalent GC dosage, which may indicate weight change occurring early in a GC course. Data from 3 trials showed that weight gain occurred, all showing that weight gain occurred early after GC initiation. Data from the Wegner's Granulomatosis Etanercept Trial (WGET) showed that weight gain occurred in the first 9 months and plateaued up to the end of follow‐up at 1 year 42. In COBRA, weight gain was significantly higher in the prednisolone group at 26 weeks but not at 56 weeks*,* perhaps indicating a plateau of weight gain, although prednisolone was tapered in most patients after 28 weeks 38, 42. In the CAMERA‐II study, body mass index (BMI) was found to increase over time, but the amount by which BMI increased diminished over time. However, the change in weight was explained by disease activity rather than treatment with GCs 43. A cohort study followed 290 prednisolone‐treated RA patients at a single clinic and showed that those treated for ≤1 year had an increase in mean weight of 2.7 kg at the last visit. Those treated for >1 year, however, had a lower mean weight at the last visit compared to baseline 44. Not all evidence supports early weight gain; an online survey found that the prevalence of weight gain increased with increasing duration of exposure to GCs; of those patients exposed <15 days, 11% reported weight gain compared to 60% of those exposed for >6 months 24.

## Weight loss following discontinuation of steroids

Only a few studies have described weight loss following discontinuation of GCs. One study followed patients in a clinical trial for a year after finishing study treatment. During the trial, patients were randomized to either 7.5 mg prednisolone or placebo and were treated for 2 years, during which time there was no significant difference between the groups in terms of weight gain. A year after finishing the study treatment, the prednisolone group had lost on average 3.1 kg, and the placebo group's weight had increased by 1.5 kg 45. Data from WGET followed a proportion of patients to 2 years or further. In those who achieved remission, weight gained during the first year was maintained. In those who had disease flares and were treated with GCs and cyclophosphamide, the mean weight gain was 1.03 kg 42.

The evidence around weight gain after GC initiation and weight loss after GC discontinuation gives some indication of effects. Some evidence exists, for example, that weight gain may occur early after GC initiation and may be linked to dose and disease activity, but clearly there is a need for more longitudinal studies to understand weight gain over time, both during and after GC treatment.

## Impact of weight gain

As mentioned above, understanding drug safety and decision‐making require consideration not just of the likelihood and extent of side effects, but also the impact that such adverse events might have on individuals. This impact in turn will affect patients’ value judgements and decision‐making. The impact of GC‐induced weight gain has received relatively little attention, despite studies reporting its importance to patients 11, 46. For example, over 40% of patients with RA considered weight gain as “most bothersome in everyday life and ascribed to glucocorticoids” compared to other listed adverse effects 46.

Weight gain can have both physical and psychological impact. Obesity is linked to an increased risk of comorbidities such as type 2 diabetes mellitus, cancer, and cardiovascular disease 47. It also has negative consequences for psychological concepts such as body image and self‐esteem. In a meta‐analysis, overweight individuals were shown to have low self‐esteem, with a stronger relationship in those perceiving themselves as heavy rather than individuals who actually are overweight 48. Body image may be regarded as a multifaceted construct composed of an individual's misconception of their own body size and an attitudinal construct concerning body dissatisfaction, body shape, and weight concerns. The increased appetite and calorie intake associated with GCs may have a negative influence on body image, as described in a study investigating food calorie intake and influence on body image in healthy volunteers 49. To our knowledge, however, there are no reported studies investigating the relationship between body image or self‐esteem specifically with drug‐induced weight gain.

## Indirect impact of weight gain

Patients experiencing undesirable side effects, such as weight gain and associated worries, may potentially alter their GC adherence. Lower adherence has the consequence of inadequate treatment efficacy to control disease, potential escalation to more intensive treatment, and waste of medication. In a cross‐sectional study of patients on long‐term (≥3 months) GC treatment, 125 of 255 patients gained >3 kg of weight over 2 years. Respondents were grouped into level of adherence (good versus poor). In all, 65% of poor adherers gained 3 kg or more, compared to 45% of good adherers 50. In the behavioral literature, the Necessity‐Concerns Framework has been consistently shown to underpin medication adherence, where those patients more skeptical of their medication, with low perceived need for their medication and high concern, are more at risk of nonadherence 51. In a cross‐sectional study of GC users, 46% (83 of 181) reported a high concern and lower perceived need for medication necessity, and of these, a third were classified as low adherers, and the remaining were classified as optimal adherers 23. Similarly, in a systemic sclerosis population, the higher the level of necessity to level of concern, the higher the medication adherence 26. However, in a population of patients experiencing adrenal inefficiency, nonadherence was associated with more GC concerns but not with necessity 32.

The concern with weight gain may well impact adherence to GCs and thereby result in poorer outcomes for patients. Future research into GCs and weight gain may allow doctors to provide patients with more detailed information about the potential extent of weight gain and weight loss after finishing GC treatment. This information may help reduce patients’ concerns and thereby increase adherence.

## Summary

Recommendations from EULAR guidelines outline the need to consider and discuss adverse effects with patients before GC treatment commences 2, 3. However, for weight gain, the extent, timing, reversibility of weight gain, and its impact are largely unknown or, at best, imprecise and thus cannot be communicated. Consequently, the clinician and therefore the patient will not be making an informed treatment decision. This review shows that weight gain is one of the GC side effects most important to patients. However, weight gain is not well measured or reported in studies, with studies often not designed to evaluate weight gain primarily, with some relying on patient self‐reporting of weight changes. A systematic review of GCs and energy intake, appetite, and weight gain across all diseases came to a similar conclusion, recommending further RCTs that are well designed and adequately powered to determine the effects of GCs on body weight 8. Evidence suggests that the psychological well‐being of patients, expressed through concerns toward GC treatment itself, the worries about weight gain, and the psychological implication of the resulting weight gain, are important issues needing attention.

Certain similarities may be drawn out and learned from extensive work carried out on antipsychotic drugs, known for causing substantial weight gain. After weight gain was identified as a side effect of antipsychotic drugs, studies have been routinely recording weight. This improved data collection has allowed greater understanding of the extent of weight gain with these drugs, and a clinically significant level of weight change has been established 52. Replication of all aspects of antipsychotic drug weight gain recording may not be possible for GCs because future large‐scale clinical trials of GCs in rheumatology are unlikely. Collection of high‐quality data regarding GC exposure, including dose and timing, in addition to longitudinal weight measurements in rheumatology cohorts, is needed. This information will provide potential important insight to examine both the rate of onset with dosage change and the speed of weight loss following discontinuation.

For the patient, having the clinician acknowledge and discuss potential weight gain before initiation of GC treatment is important. Weight monitoring during GC treatment and addressing patient concerns together with better information about the likelihood and extent of weight gain and potential loss after a period of GC treatment cessation may well improve the psychological well‐being of patients. These improvements may, in turn, lower uncertainty and increase confidence in GC use by patients and maintain persistence over the GC course to ultimately improve the control of disease progression.

## Author Contributions

All authors were involved in drafting the article or revising it critically for important intellectual content, and all authors approved the final version to be submitted for publication. Dr. Dixon had full access to all of the data in the study and takes responsibility for the integrity of the data and the accuracy of the data analysis.

### Study conception and design

Morgan, Dixon.

### Acquisition of data

Morgan, Costello.

### Analysis and interpretation of data

Morgan, Costello, Ray, Dixon.
