# Supplementary material for: How Do Glucocorticoids Used in Rheumatic Disease Affect Body Weight? A Narrative Review of the Evidence
Source: Arthritis Care Res (Hoboken). 2020 Mar 27;72(4):489–97. doi: 10.1002/acr.23879 (PMC7155058; doi:10.1002/acr.23879)

**Appendix 1**

**Search strategy**

- English language articles including all years
- Broad search terms for relevant GC use in RA and weight change:

| **Patient population**  Rheum* arthrit*  Rheum* polyarthri*  Inflammatory arthrit*  Inflammatory polyarthrit* | **Exposure:**  Glucocorticoid  Corticosteroid  Glucocorticosteroid  Cortisone  Hydrocortisone  Prednisolone  Prednisone  Betamethasone  Deflazacort  Dexamethasone | **Outcome**  Adverse event  Adverse effect  Adverse outcome  Side effect  Weight change  Body weight  Weight gain  Weight loss  Risk  Benefit |
| --- | --- | --- |
| **Include SIGN filters for trials and cohort studies** | | |

- Using Web of Science and Cochrane reviews
- All relevant papers reviewed including appendices for additional withdrawal details in trials
- Additional referenced papers within retrieved papers reviewed for potential inclusion

**1) Web of science literature search terms**

Indexes=BKCI-S, SSCI, BKCI-SSH, SCI-EXPANDED, A&HCI, IC, CPCI-SSH, CPCI-S, CCR-EXPANDED Timespan=All years

| **Set** | **Results** |  |
| --- | --- | --- |
| # 51 | 26 | #50 AND #37 |
| # 50 | 384 | #49 AND #4 |
| # 49 | 17,051 | #48 AND #36 AND #1 |
| # 48 | 2,472,412 | #47 OR #42 |
| # 47 | 2,230,983 | **TOPIC:** (risk OR benefit) |
| # 46 | 564 | #43 AND #37 |
| # 45 | 14 | #44 AND #37 |
| # 44 | 204 | #43 AND #4 |
| # 43 | 7,275 | #42 AND #36 AND #1 |
| # 42 | 339,553 | TS=((advers$ or side$) near/2 (outcome$ or effect$ or event$)) |
| # 41 | 23 | #37 AND #35 AND #4 AND #1 |
| # 40 | 51 | #37 AND #36 AND #4 AND #1 |
| # 39 | 113 | #38 AND #37 AND #4 AND #1 |
| # 38 | 8,090,975 | TS=(human* OR patient* OR people* OR participant* OR volunteer* OR adult* OR women* OR athlete*) |
| # 37 | 121,968 | ts=(((rheum* OR inflammatory) near/1 (arthrit* OR polyarthrit*)) NOT juvenile) |
| # 36 | 1,803,451 | #35 OR #24 |
| # 35 | 1,165,930 | #34 OR #33 OR #32 OR #31 OR #30 OR #29 OR #28 OR #27 OR #26 OR #25 |
| # 34 | 189,634 | ts="cross sectional" |
| # 33 | 189,638 | ts=cross-sectional |
| # 32 | 257,314 | ts=retrospective |
| # 31 | 260,153 | ts=longitudinal |
| # 30 | 55,621 | TS=(observational near/1 study) OR TS=(observational near/1 studies) |
| # 29 | 42,462 | TS=("follow up" near/1 study) OR TS=("follow up" near/1 studies) |
| # 28 | 102,989 | TS=(cohort near/1 study) OR TS=(cohort near/1 studies) |
| # 27 | 246,747 | TS=cohort studies |
| # 26 | 247,578 | TS=case control studies |
| # 25 | 36,803 | TS=epidemiologic studies |
| # 24 | 717,917 | #19 NOT #23 |
| # 23 | 835,331 | #22 OR #21 OR #20 |
| # 22 | 26,527 | **TOPIC:** (historical article) |
| # 21 | 132,876 | **TOPIC:** (letter) |
| # 20 | 677,749 | **TOPIC:** (case report) |
| # 19 | 741,070 | #18 OR #14 |
| # 18 | 491,515 | #17 OR #16 OR #15 |
| # 17 | 182,871 | **TOPIC:** (placebo$) |
| # 16 | 193,018 | **TOPIC:** (((singl* OR doubl* OR treb* OR tripl*) near/3 (blind* OR mask*))) |
| # 15 | 262,072 | **TOPIC:** (clinical near/1 trial$) |
| # 14 | 613,518 | #13 OR #12 OR #11 OR #10 OR #9 OR #8 OR #7 OR #6 |
| # 13 | 15,381 | **TOPIC:** (multicentre study) |
| # 12 | 174,480 | **TOPIC:** (controlled clinical trial) |
| # 11 | 67,846 | **TOPIC:** (clinical trial, phase) |
| # 10 | 419,558 | **TOPIC:** (clinical trial) |
| # 9 | 15,503 | **TOPIC:** (Single Blind Method) |
| # 8 | 59,685 | **TOPIC:** (Double Blind Method) |
| # 7 | 5,236 | **TOPIC:** (Random Allocation) |
| # 6 | 258,912 | **TOPIC:** (randomi$ed controlled trial$) |
| # 5 | 260,440 | TS=((weight) near/2 (gain OR loss OR change$ OR body)) NOT TS=fetal weight NOT TS=birth weight |
| # 4 | 252,559 | TS=((weight) near/1 (gain OR loss OR change$ OR body)) NOT TS=fetal weight NOT TS=birth weight |
| # 3 | 275,521 | **TOPIC:** (((weight) near/2 (gain OR loss OR change$ OR body))) |
| # 2 | 266,886 | **TOPIC:** (((weight) near/1 (gain OR loss OR change$ OR body))) |
| # 1 | 217,852 | **TOPIC:** (glucocorticoid$ OR corticosteroid$ OR glucocorticosteroid$ OR cortisone OR hydrocortisone OR prednisone OR *prednisolone OR betamethasone OR deflazacort OR dexamethasone) |


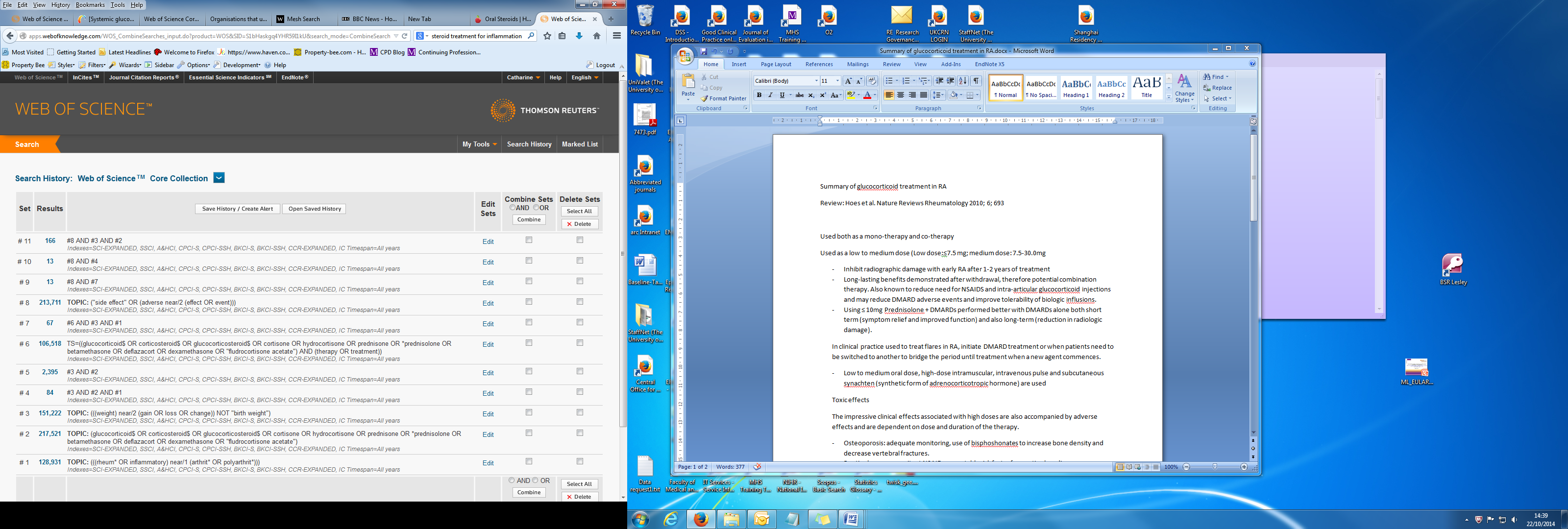


**2) Cochrane review search terms:**


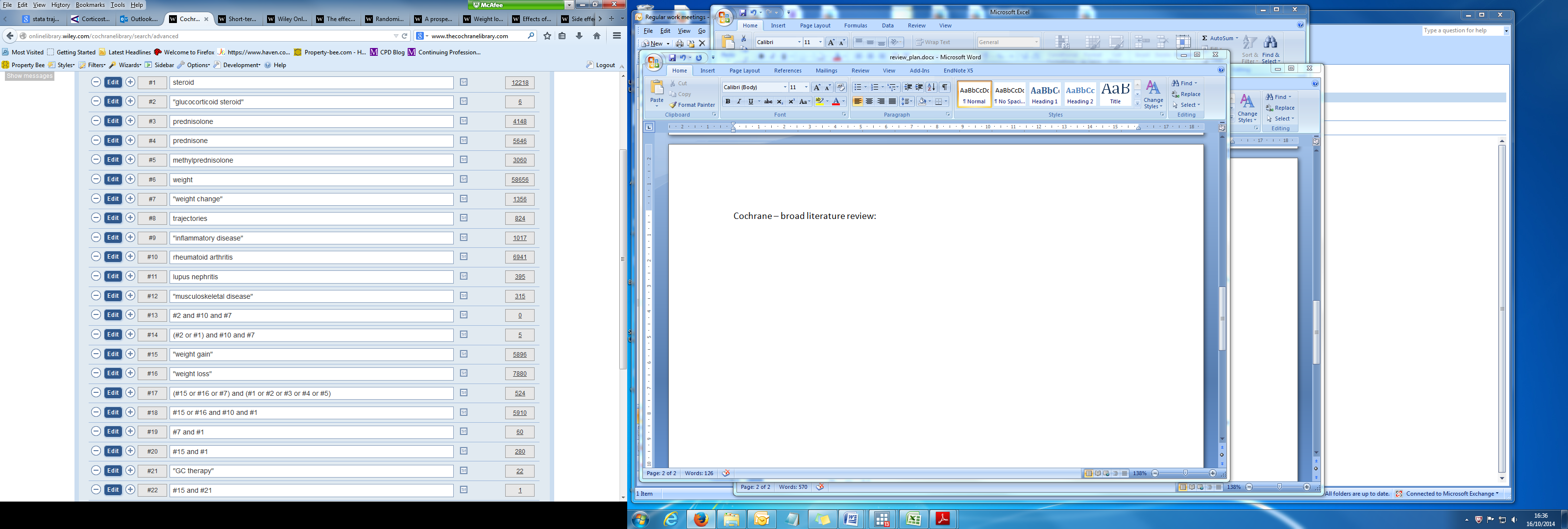

Supplement: Supplementary file 1 [file ACR-72-489-s001.docx]
